# Cancer: a “stem-cell” disease?

**DOI:** 10.1186/1475-2867-13-40

**Published:** 2013-05-06

**Authors:** Shi-Ming Tu

**Affiliations:** 1 Department of Genitourinary Medical Oncology, Unit 1374, The University of Texas MD Anderson Cancer Center, 1155 Pressler Street, Houston, TX 77030-3721, USA

**Keywords:** Cancer stem cells, Stem-ness, Microenvironment, Personalized care, Paradigm shift

## Abstract

**Background:**

Nowadays, we believe that cancer is a genetic disease. We focus on the genetic targets and epigenetic changes in a tumor. Remarkably, many crucial signal pathways in a malignant cell involve “stem-ness” genes. The prevalence of stem-ness in cancer suggests that cancer has a stem-cell origin and is a stem-cell disease.

**Presentation of the hypothesis:**

The observation that many innate stem-ness properties are easily interchangeable with malignant hallmarks needs to be further elucidated. There appears to be a malignant potential in every stem cell and a stem cell potential in every malignant cell. I hypothesize that cancer is a stem-cell disease rather than a genetic disease.

**Testing the hypothesis:**

We will use homeobox genes to endow a certain progenitor cell with specific stem-ness properties and confer different stem-cell phenotypes to the particular cell type in a hierarchical manner. We will demonstrate that an earlier homeobox gene plus a genetic defect (such as Pten loss) tend to form a more virulent tumor, while a later homeobox gene plus the same genetic defect tend to express a more indolent phenotype. Importantly, we will show that in clinically relevant cancer subtypes, those with worse clinical outcomes may paradoxically harbor fewer genetic mutations than those with better outcomes do.

**Implications of the hypothesis:**

The recognition that cancer is a stem-cell disease will instigate major paradigm shifts in our basic understanding of cancer. Many fundamental principles of oncology, such as multistep carcinogenesis, need to be reconciled. The realization that cancer is a stem-cell disease will also have profound clinical implications on personalized care. Many aspects of our current clinical trials need to be reevaluated.

## Background

**Figure 1:**
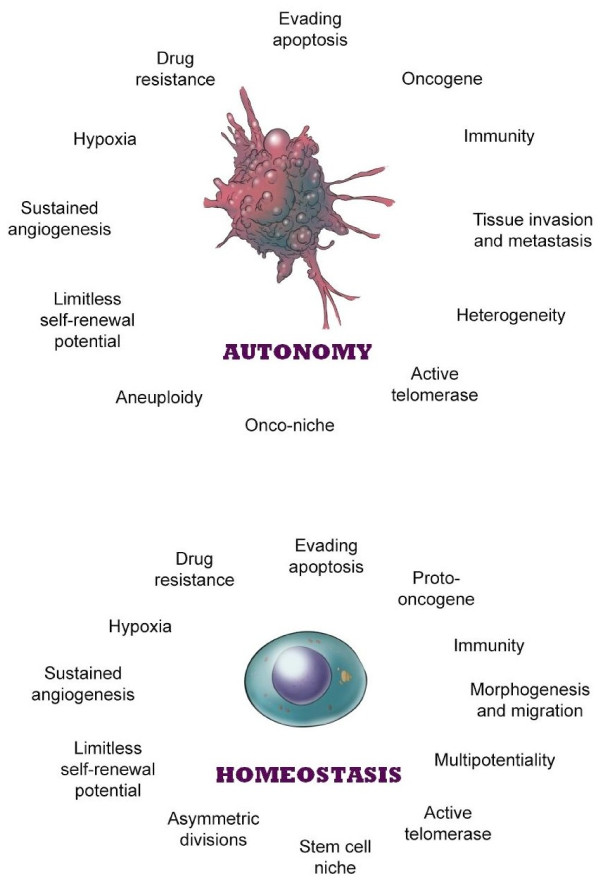
**Cancer and stem cells.** Cancer cells (top panel) and stem cells (bottom panel) are like mirror images of each other. There appears to be malignant potential in every stem cell and stem cell potential in every malignant cell. Reprinted with permission from Springer [2].

Despite our increased knowledge and better understanding of cancer, we are still quite clueless about its exact origin. Although genetic and epigenetic mechanisms play a critical role in the development of cancer, their relevance depends to a large extent on the right cellular context and the microenvironment. Considering all the various aspects of cancer, we notice a common thread that unites cancer, namely “stem-ness.” Stem-ness accounts for the dormancy, regeneration, heterogeneity, immunity, and metastasis of cancer. It seems that stem-ness is not only intrinsic but also integral to cancer (Figure 1) [1,2].

The idea that cancer has a stem-cell origin is not new. In 1863, Rudolf Virchow first promulgated a stem-cell theory of cancer [3]. In 1959, Barry Pierce performed seminal experiments that reignited interest in this field [4]. Recently, many prominent investigators have provided scientific evidence that validates, if not proves, the theory of a stem-cell origin of cancers [2]. However, to my knowledge, no one has yet hypothesized that cancer is a stem-cell disease.

An indispensable component of the stem-cell theory is the interplay between cancer and the microenvironment. In many respects, a stem cell is inseparable from its stem-cell niche, just like a cancer cell is from its onco-niche. This is best illustrated by classic experiments in which normal stem cells derived from the genital ridge formed teratomas when placed in an exogenous site [5], whereas teratocarcinoma cells formed parts of a normally developing mouse when implanted into the inner cell mass of a blastocyst [6]. Further, Rous sarcoma virus did not induce sarcomas in chicken embryos [7], and B16 murine melanoma cells failed to form tumors in the embryonic skin [8].

Houghton et al. [9] performed a pivotal experiment that proved the stem-cell theory of cancer. They demonstrated that bone marrow–derived stem cells traveled to the *Helicobacter*-infected stomach of C57BL/6 mice and were the source of gastric carcinoma. Recently, many other investigators have devised ingenious experiments to elucidate the identity of cancer-initiating cells with stem-ness properties at well-known premalignant sites [10,11] and in disparate malignant tumors [12-14].

## Controversy

It is important to emphasize that the idea of cancer being a stem-cell disease goes beyond the idea of cancer stem cells (CSCs). It may be the key to resolve the controversy of whether CSCs are derived from stem cells, acquire stemness features, or merely mimic stem cells [2].

A reservation about the existence of CSCs concerns experiments showing that CSCs and non-CSCs (ie, differentiated cancer cells) are interchangeable, suggesting that CSCs may be a transient rather than a unique entity [15,16]. However, it is plausible that unknown populations of CSCs not detectable by currently available “stem-cell markers” (e.g., CD44^+^, ALDH) are present in those assays. Otherwise, why would a small percentage of non-CSCs (i.e., 0.4/300 of HME-flopc-CD44^lo^ cells) form mammospheres [15]? Why would sorted CSCs fail to stably propagate, and yet there was a constant proportion of CSCs within the transformed population [16]? Interestingly, Zhao et al. demonstrated that induced pluripotent stem cells (iPSCs) from somatic cells reprogrammed with stemness factors have stem-cell features. But iPSCs are not embryonic stem cells (ESCs). Unlike teratomas arising from ESCs, tumors derived from iPSCs are duly recognized and rejected by the immune system of syngeneic immunocompetent mice [17].

Another reservation about the existence of CSCs relates to the role of the microenvironment and how the stroma affects initiation or progression of cancer [18,19]. Hence, epigenetic alterations in the stroma are sufficient to initiate formation of carcinoma and precede the development of any mutations in adjacent epithelial cells [18]. But the fact remains that the involved epithelial cells affected by the stroma have stem-ness characteristics, because they have the capacity to regenerate whole organ systems [19]. Interestingly, Reticker-Flynn et al. reported a novel way to block metastasis by focusing on specific cellular interactions rather than particular gene mutations [20]. They demonstrated that metastatic cells selectively associate with fibronectin in combination with galectin-3, galectin-8, or laminin (which constitute the stem-cell niche) through α3β1 integrin (which is expressed by CSCs).

It is ironic that the missing piece in the genetic theory of cancer may actually be the centerpiece in the stem-cell theory of cancer.

## Presentation of the hypothesis

I hypothesize that cancer is a stem-cell disease. I propose that our traditional view of cancer, that cancer has a genetic origin and is a genetic disease, is incomplete. Although mutations do cause cancer, the idea that we need to fix a particular mutation to *cure* cancer is fallible. For example, prostate cancer may contain 3,866 somatic base mutations per tumor and 90 chromosomal aberrations per genome [21]. Perhaps we only need to focus on the so-called “driver genes.” But many of these driver genes actually have stem-ness properties [22].

The pervasive idea that cancer may arise from any cells in the body is also untrue. For example, innumerable key oncogenic defects (e.g., *p53*, *PTEN*) are also found in nonmalignant cells [23,24]. If accumulation of mutations causes progression of cancer, and the rate of mutation is relatively low in humans (i.e., <200 new mutations per diploid genome per generation) [25], then there is insufficient time for the mutations to occur and accumulate in a somatic cell with a limited life span (e.g., skin, 30 days; gut, 3 days) [26]. Perhaps “genetic instability” can account for this discrepancy. But genetic instability is also traceable to a vital stem-ness trait, namely asymmetric division [27].

## Testing the hypothesis

According to the stem-cell theory of cancer, the type of cell in which a genetic mutation occurs is just as important as (if not more so than) the mutation itself during carcinogenesis. I hypothesize that the same mutations which affect earlier cancer-initiating cells may also affect later progenitor cells in a stem-cell hierarchy. Paradoxically, earlier cancer-initiation cells that form more virulent tumors may require fewer mutations to become malignant than later progenitor cells that form more indolent tumors do, because their inherent stem-ness obviates the need to acquire more mutations. After all, many stem-ness properties are also potential malignant hallmarks (Figure 1).

We can test this hypothesis by using homeobox genes, which provide a particular progenitor cell with specific stem-ness properties that confer different stem-cell phenotypes to the same cell in a hierarchical manner. Hence, an earlier homeobox gene plus a genetic defect (such as Pten loss) in a particular cell type may form a more heterogeneous tumor that metastasizes more widely, while a later homeobox gene plus the same genetic defect in the same cell type is likely to express a more restricted and indolent phenotype. Importantly, we can show that in clinically relevant cancer subtypes, those with worse clinical outcomes tend to harbor fewer mutations than those with better outcomes do.

## Implications of the hypothesis

The recognition that cancer is a stem-cell disease will instigate major paradigm shifts in our basic understanding of cancer. Many fundamental principles of oncology need to be reconsidered and reconciled.

For example, a paradigm shift is in order regarding dedifferentiation of cancer. Does a cancer cell become dedifferentiated from a mature differentiated cell, or does it merely reveal its undifferentiated stem-cell features? Also, if a late event like metastasis occurs early during carcinogenesis, it contradicts our classic model of multistep carcinogenesis, which assumes that cancer becomes more metastatic as it acquires and accumulates increasing numbers and types of genetic mutations. The idea that cancer is a stem-cell disease rather than a genetic disease may alter the entire landscape of cancer and revamp the whole groundwork of oncology.

The realization that cancer is a stem-cell disease has profound clinical implications on cancer care, including personalized care. Many aspects of our current clinical trials need to be revisited and reevaluated.

I anticipate that personalized care based on a specific cellular entity would be more efficacious than that which is based solely on a specific genetic mutation within it. After all, a common link between the cancer-initiating cells with stem-ness properties and the mutations they contain is the cell of origin. Consequently, when we target an aberrant cancer-initiating cell with its whole package of genetic mutations rather than the individual mutations themselves, we are treating a whole system of intracellular, intercellular, and microenvironmental pathways or networks.

Currently, our clinical trials are not designed to assess the therapeutic effects and potential benefits of agents that target CSCs or cancer-initiating cells with stem-ness properties. We need to be cognizant that treatments that target CSCs may provide delayed therapeutic benefits, because of a lag in the detection of perceptible clinical improvement. Otherwise, promising treatments could be prematurely abandoned unless and until we develop therapeutic strategies to account for the presence of CSCs and response criteria to monitor the effects of therapy on them. When clinical trials are designed to target appropriate patient populations with specific cancer subtypes and relevant CSCs, we predict that the overall patient survival time may improve for a few years, rather than just a few months. When we conduct clinical trials on the basis of a correct and pertinent cancer hypothesis, we save money, time, and lives.

## Abbreviations

CSC: Cancer stem cell.; Cancer cells: a generic term for malignant cells that include both cancer stem cells and differentiated cancer cells; Cancer targets: targets for cancer biomarkers or anti-cancer therapy used as an umbrella term to describe molecular and/or genetic alterations in cells – some of which may be good targets for cancer biomarkers or anti-cancer therapy and others not; Dedifferentiation: conversion of a differentiated phenotype to an undifferentiated one during malignant transformation; Onco-niche: a specialized microenvironment that “houses” cancer cells just as a stem-cell niche that houses stem cells; Progenitor stem cells: relatively more mature stem cells or progenitor cells with stem-cell properties that might be the actual targets of tumorigenic transformation also defined in this article as the origin of cancer-initiating cells; Stem cells: undifferentiated cells with self-renewal and differentiating capacities; Stem-ness: properties or potential that make a stem cell, including effects from the stem-cell niche.

## Competing interests

This article contains ideas from the author’s book, *Origin of Cancers: Clinical Perspectives and Implications of a Stem-Cell Theory of Cancer*. Proceeds from this book are dedicated to cancer research.

## Authors’ contributions

S-MT is the sole author of this article.

## Authors’ information

S-MT is Clinical Professor in the Department of Genitourinary Medical Oncology at The University of Texas MD Anderson Cancer Center in Houston, Texas. He earned his undergraduate degree from the Johns Hopkins University, Baltimore, Maryland, and his medical degree from Washington University in St. Louis, Missouri. His article, “Stem-cell origin of metastasis and heterogeneity in solid tumours,” was published in *Lancet Oncology* in 2002, and his book, *Origin of Cancers: Clinical Perspectives and Implications of a Stem-Cell Theory of Cancer*, by Springer in 2010.

## References

[B1] Tu S-M Lin S-H Logothetis CJ Stem-cell origin of metastasis and heterogeneity in solid tumours Lancet Oncol 2002 3 508 513 10.1016/S1470-2045(02)00820-3 12147437

[B2] Tu S-M Rosen ST Origin of cancers. Clinical perspectives and implications of a stem-cell theory of cancer Cancer treatment and research; vol. 154 2010 New York: Springer 10.1007/978-1-4419-5968-3_1 20645140

[B3] Virchow R Fitzgerald PJ Die krankhaften geschwulste [in German]. 3 vols. Berlin: a hirschwald, 1863–1865 From demons and evil spirits to cancer genes 2000 Washington DC: American Registry of Pathology

[B4] Pierce GB Dixon FJ Jr Testicular teratomas I. Demonstration of teratogenesis by metamorphosis of multipotential cells Cancer 1959 12 573 583 10.1002/1097-0142(195905/06)12:3<573::AID-CNCR2820120316>3.0.CO;2-M 13652104

[B5] Stevens LC Experimental production of testicular teratomas in mice Proc Natl Acad Sci USA 1964 52 654 661 10.1073/pnas.52.3.654 14212538 PMC300322

[B6] Illmensee K Russell LB Reversion of malignancy and normalized differentiation of teratocarcinoma cells in mammals Genetic mosaics and chimeras in mammals. Basic life science series; vol 12 1978 New York: Plenum 3 25 10.1007/978-1-4684-3390-6_1 378217

[B7] Dolberg DS Bissell MJ Inability of Rous sarcoma virus to cause sarcomas in the avian embryo Nature 1984 309 552 556 10.1038/309552a0 6203040

[B8] Gerschenson M Graves K Carson SD Wells RS Pierce GB Regulation of melanoma by the embryonic skin Proc Natl Acad Sci USA 1986 83 7307 7310 10.1073/pnas.83.19.7307 3463969 PMC386705

[B9] Houghton J Stoicov C Nomura S Gastric cancer originating from bone marrow–derived cells Science 2004 306 1568 1571 10.1126/science.1099513 15567866

[B10] Wang X Ouyang H Yamamoto Y Residual embryonic cells as precursors of a Barrett’s-like metaplasia Cell 2011 145 1023 1035 10.1016/j.cell.2011.05.026 21703447 PMC3125107

[B11] Herfs M Yamamoto Y Laury A A discrete population of squamocolumnar junction cells implicated in the pathogenesis of cervical cancer Proc Natl Acad Sci USA 2012 109 10516 10521 10.1073/pnas.1202684109 22689991 PMC3387104

[B12] Driessens G Beck B Caauwe A Simons BD Blanpain C Defining the mode of tumour growth by clonal analysis Nature 2012 488 527 530 10.1038/nature11344 22854777 PMC5553110

[B13] Schepers AG Snippert HJ Stange DE Lineage tracing reveals Lgr5^+^ stem cell activity in mouse intestinal adenomas Science 2012 337 730 735 10.1126/science.1224676 22855427

[B14] Chen J Li Y Yu T-S A restricted cell population propagates glioblastoma growth after chemotherapy Nature 2012 488 522 526 10.1038/nature11287 22854781 PMC3427400

[B15] Chaffer CL Brueckmann I Schell C Normal and neoplastic nonstemcells can spontaneously convert to a stem-cell state Proc Natl Acad Sci USA 2011 108 7950 7955 10.1073/pnas.1102454108 21498687 PMC3093533

[B16] Iliopoulos D Hirsch HA Wang G Struhl K Inducible formation of breast cancer stem cells and their dynamic equilibrium with non-stem cancer cells via IL-6 secretion Proc Natl Acad Sci USA 2011 108 1397 1402 10.1073/pnas.1018898108 21220315 PMC3029760

[B17] Zhao T Zhang ZN Rong Z Xu Y Immunogenicity of induced pluripotent stem cells Nature 2011 474 212 215 10.1038/nature10135 21572395

[B18] Zong Y Huang J Sankarasharma D Stromal epigenetic dysregulation is sufficient to initiate mouse prostate cancer via paracrine Wnt signaling Proc Natl Acad Sci USA 2012 109 E3395 E3404 10.1073/pnas.1217982109 23184966 PMC3528570

[B19] Xin L Ide H Kim Y Dubey P Witte ON In vivo regeneration of murine prostate from dissociated cell populations of postnatal epithelia and urogenital sinus mesenchyme Proc Natl Acad Sci USA 2003 100 Suppl 1 11896 11903 12909713 10.1073/pnas.1734139100 PMC304104

[B20] Reticker-Flynn NE Braga Malta DF Winslow MM A combinatorial extracellular matrix platform identifies cell-extracellular matrix interactions that correlate with metastasis Nat Commun 2012 3 1122 10.1038 23047680 10.1038/ncomms2128 PMC3794716

[B21] Berger MF Lawrence MS Demichelis F The genomic complexity of primary human prostate cancer Nature 2011 470 214 220 10.1038/nature09744 21307934 PMC3075885

[B22] Johnson RA Wright KD Poppleton H Cross-species genomics matches driver mutations and cell compartments to model ependymona Nature 2010 466 632 636 10.1038/nature09173 20639864 PMC2912966

[B23] Firestein GS Echeverri F Yeo M Zvaifler NJ Green DR Somatic mutations in the p53 tumor suppressor gene in rheumatoid arthritis synovium Proc Natl Acad Sci USA 1997 94 10895 10900 10.1073/pnas.94.20.10895 9380731 PMC23522

[B24] Pap T Franz JK Hummel KM Jeisy E Gay R Gay S Activation of synovial fibroblasts in rheumatoid arthritis: lack of expression of the tumor suppressor PTEN at sites of invasive growth and destruction Arthritis Res 2000 2 59 64 10.1186/ar69 11219390 PMC17804

[B25] Nachman MW Crowell SL Estimate of the mutation rate per nucleotide in humans Genetics 2000 156 297 304 10978293 10.1093/genetics/156.1.297 PMC1461236

[B26] Sell S Pierce GB Maturation arrest of stem cell differentiation is a common pathway for the cellular origin of teratocarcinomas and epithelial cancers Lab Invest 1994 70 6 22 8302019

[B27] Morrison SJ Kimble J Asymmetric and symmetric stem-cell divisions in development and cancer Nature 2006 441 1068 1074 10.1038/nature04956 16810241

